# Tomographic findings in patients with COVID-19 according to evolution of the disease

**DOI:** 10.1186/s43055-020-00329-5

**Published:** 2020-10-27

**Authors:** Axel Quispe-Cholan, Yoselyn Anticona-De-La-Cruz, Marco Cornejo-Cruz, Oswaldo Quispe-Chirinos, Veronica Moreno-Lazaro, Edward Chavez-Cruzado

**Affiliations:** 1 Human Medical School, Universidad Privada Antenor Orrego, Trujillo, Peru; 2 Resomag Radiology Service, Trujillo, Peru; 3 Tomonorte Radiology Service, Trujillo, Peru

**Keywords:** Coronavirus infections, X-ray computed tomography, Diagnosis, Disease progression

## Abstract

**Background:**

The tomographic findings in COVID-19, its classification, a brief overview of the application of artificial intelligence, and the stages during the course of the disease in patients with moderate COVID-19

**Main body:**

Chest CT allows us to follow the course of COVID-19 in an objective way; each phase has characteristic imaging findings and, consequently, takes the corresponding measures.

A search was made in the PubMed database with the keywords extracted from the DeCs and the combinations of these. Only articles published between December 2019 and June 2020 were included. The search was limited to the English language.

**Conclusions:**

CT serves to monitor the course of the disease since it assesses the severity of lung involvement. The most frequent finding is bilateral ground glass opacities with a subpleural distribution. The progression occurs in two phases: one slow and one fast. At discharge, the patient may have ground glass opacities or areas that will later become fibrosis, leaving sequelae for life.

## Background

At the end of 2019, 27 cases of pneumonia of unknown origin were identified in the city of Wuhan, China [1, 2]. The causative agent of this disease was identified from nasopharyngeal swab samples by the Centers for Disease Control and Prevention (CDC), on January 7, 2020; it was called severe acute respiratory syndrome coronavirus type 2 (SARS-CoV-2). The disease was named COVID-19 by the World Health Organization [3]. At the moment, the diagnosis is based on clinical findings, the patient’s epidemiological history, findings on chest computed tomography (CT), and reverse transcriptase polymerase chain reaction (RT-PCR), the last being the “gold standard” [4]. Chest CT allows us to monitor the course of the disease in an objective way; each phase has characteristic imaging findings and, consequently, takes the corresponding measures. This review seeks to document the tomographic findings in COVID-19, its classification, a brief overview of the application of artificial intelligence, and the stages during the course of the disease in patients with moderate COVID-19 [5–7].

A search was made in the PubMed, Scopus, and Web of Sciences database with the keywords extracted from the DeCs and the combinations of these. Only articles published between December 2019 and June 2020 were included. The search was limited to the English language.

## Main text

### COVID-19 and radiology

In the current COVID-19 pandemic, radiology plays a fundamental role in patient management. Among the available options we have, X-rays, ultrasound, and CT of the chest; each with its advantages and disadvantages; however, chest CT is the standard in imaging studies of pneumonia [8]. Chest CT is a non-invasive radiological examination that involves the use of X-rays at different angles around the patient’s chest to produce cross-sectional images [9, 10]. As COVID-19 spreads across the world, there is increasing interest in the role of chest CT in screening, diagnosis, and management of patients with suspected or confirmed COVID-19 infection. The American College of Radiology recommends that chest CT should be used with discretion and reserved for hospitalized and symptomatic patients with specific clinical indications for CT. Likewise, it indicates that it should not be used as a first-line diagnostic test [11, 12]. Chest CT is used to assess the severity of the lung affected by COVID-19 [13, 14]. However, in some situations such as a shortage of RT-PCR tests in emergency situations, it can be considered as a diagnostic option. This is because it provides, at initial stages, a higher sensitivity to molecular tests also due to the speed with which the images are obtained, and thus reduce the false negative rate [15]. With this benefit, the appropriate isolation measures can be taken as well as applying the appropriate management [16, 17].

### CT: imagenological findings/classification of findings

COVID-19 has a wide spectrum of tomographic characteristics reported in various studies [18, 19]. Chest radiographs have little diagnostic value in the early stages, while CT findings may be present even before the onset of symptoms [18]. Frequent features such as ground glass opacities with or without consolidated anomalies are considered compatible with viral pneumonia [20–22]. If these findings are added multifocality, a predominant distribution in subpleural and peribronchovascular regions, they can be considered a COVID-19 pattern [23]. Currently, there is a greater publication of studies on imaging features focused on CT in COVID-19. Among the publications, a consensus study with the participation of the Chinese Society of Radiology stands out, where it is proposed to divide the findings into two large groups: frequent and infrequent (Table 1); the Radiological Society of North America classifies the findings into four categories: typical, indeterminate, atypical, and unrelated findings (Table 2) [24–26]; and the Radiological Society of the Netherlands classifies the findings according to the level of suspicion of pulmonary involvement by COVID-19, for this they use a Classification System of Reports and Data in COVID-19 (CO-RADS) (Table 3). This system has values from 0 to 6 [27, 28].

**Table 1:** Classification according to the Chinese Society of Radiology

Category	Description
Frequent findings	Ground glass opacities, consolidations, increased lung density of vascular structures, crazy cobblestone pattern, and reverse halo sign. The lesions obey a bilateral, peripheral, peripheral distribution, with ill-defined and asymmetric borders.
Rare findings	Pleural effusion, changes in the caliber of the vessels, traction bronchiectasis, tumors, and thoracic lymphadenopathy.

**Table 2:** Classification according to the American Society of Radiology

Category	Description
Typical findings	Peripheral and bilateral ground glass opacities accompanied or not by consolidations or “crazy cobblestone” pattern; round morphology multifocal ground glass opacities with or without consolidations or “crazy cobblestone” pattern; and reverse halo sign.
Indeterminate findings	Absence of typical findings along with multifocal, diffuse, perihilar, unilateral, non-peripheral ground glass opacities, lacking rounded morphology with or without associated consolidations.
Atypical findings	Absence of typical and indeterminate findings plus segmental consolidations or those that affect an isolated single lobe without ground glass opacities; discrete small nodules (centrilobular or “budding tree” pattern); and pulmonary cavitation or mild increase in thickness of the septum with pleural effusion.
Unrelated findings	Findings not related to COVID-19.

**Table 3:** Classification according to the Radiological Society of the Netherlands

CO-RADS	Suspicion level	Description
0		Findings do not correspond to any category.
1	Very low	Mild or severe emphysema, nodules around fissures, lung tumors, or fibrosis.
2	Low	Findings of a budding tree pattern, bronchiectasis, centrilobular nodular pattern, lobar or segmental consolidations, and lung cavitation.
3	Indeterminate	Perihilar ground glass opacities, homogeneous and extensive ground glass pattern with or without preservation of some secondary lung lobes, or ground glass together with thickening of the interlobular septum with or without pleural effusion.
4	High	Findings are not in contact with the visceral pleura or are located unilaterally, have a predominant peribronchiovascular distribution, or overlap with severe pre-existing lung abnormalities.
5	Very high	Ground glass opacities, with or without consolidations, in regions close to the surface of the visceral pleura including fissures and present a bilateral multifocal distribution. Presence of a “crazy cobblestone” pattern, reverse halo sign, subpleural bands and thickening of the vascular network within the pulmonary anomalies.
6		Findings of a patient with positive RT-PCR.

## Classification according to the Chinese Radiology Consensus

### Frequent

In the study with the largest population to date (940 people), ground glass opacities and mixed attenuation pattern are the most frequent findings [18]. Ground glass opacities present as a slight increase in lung density. With visibility of the vascular structures, they are due to pathological changes such as serous and fibrous exudates as well as vascular congestion of the septa and edema [29–31]. Subsequently, these areas grow, cover a larger area, and become denser. To this are added consolidations with an air bronchogram sign. In consolidation, there is an increase in parenchymal density; its extension may be acinar to encompass a whole lung lobe. Consolidations are more prevalent in the elderly. The lung lesions are bilateral; peripheral, with poorly defined edges; asymmetric, with nodular; patched; or confluent morphology, mainly in the caudal regions of the lung and the subpleural dorsal area [32, 33]. The result of the thickening of the interlobular and intralobar septum is the “crazy paving” pattern, all of which is a reflection of interstitial damage [34]. Reverse halo sign, defined as ground glass opacity zone surrounded by peripheral consolidation, and the halo sign, defined by a consolidation zone in which a ground glass opacity zone converges, are two findings closely related to the pneumonia established by COVID-19.

### Infrequent

Less frequent findings include pleural effusion, changes in vessel caliber, traction bronchiectasis, and thoracic lymphadenopathy [35]. Kong et al. reported the presence of cavitation caused by the disease in their study [36]. As the studies progress, and new information emerges, not so common findings will appear.

## Classification according to the Radiological Society of North America

### Typical

In the literature, these findings are documented as having high prevalence and specificity for COVID-19 pneumonia. This CT findings including periferal and biateral ground glass opacities accompanied or not by consolidations or “crazy paving” pattern, multifocal ground glass opacities with rounded morphology with or without consolidations or “crazy cobblestone” pattern, and the inverse halo sign along with other findings of organizing pneumonia [37, 38].

### Indeterminate

Non-specific findings for pneumonia are part of this group. It is defined by the absence of typical findings plus the presence of multifocal, diffuse, perihilar, unilateral, non-peripheral ground glass opacities, lacking rounded morphology with or without associated consolidations. This last component can be replaced by very small ground glass opacities without peripheral distribution or rounded morphology.

### Atypical

These are findings whose presence in documented studies is not common or is not reported. In order to classify them in this group, they must not be part of the 2 previous groups, and to this must be added the presence of segmental consolidations or that affect a single lobe in isolation without ground glass opacities; discrete small nodules (centrilobular or in a “budding tree” pattern); and pulmonary cavitation or mild increase in the thickness of the septum with pleural effusion [39, 40].

### Unrelated

The findings correspond to other suggestive pneumonia.

## Classification according to the Radiological Society of the Netherlands

### CO-RADS 0

It corresponds to findings that cannot be assigned to the other categories because the examination was incomplete or due to the presence of artifacts caused by breathing or coughing at the time of the study.

### CO-RADS 1

It implies a very low level of suspicion of pulmonary involvement by COVID-19 and indicates a non-infectious pathology. To this group belong the findings of mild or severe emphysema, nodules around the fissures, lung tumors, or the presence of fibrosis.

### CO-RADS 2

It implies a low level of suspicion of pulmonary involvement by COVID-19. The images are typical of other infectious diseases such as bronchitis, infectious bronchiolitis, bronchopneumonia, lobar pneumonia, and lung abscess. The findings of a budding tree pattern, bronchiectasis, centrilobular nodular pattern, lobar or segmental consolidations, and lung cavitation are present.

### CO-RADS 3

It implies indeterminate findings of pulmonary involvement by COVID-19, but that can be found in other viral pneumonias and non-infectious pathologies. This includes perihilar ground glass opacities, a homogeneous and extensive ground glass pattern with or without preservation of some secondary pulmonary lobes, or ground glass along with thickening of the interlobular septum with or without pleural effusion in the absence of typical findings.

### CO-RADS 4

It implies a high level of suspicion of pulmonary involvement by COVID-19 but whose findings may overlap to some level with other viral pneumonias. The findings are similar to CO-RADS 5 but are not in contact with the visceral pleura or are located strictly in a single lung, have a predominant peribronchiovascular distribution, or overlap with severe pre-existing lung abnormalities.

### CO-RADS 5

It implies a very high level of suspicion for lung involvement by COVID-19. These are the typical findings of the disease, ground glass opacities are present, with or without consolidations, in regions close to the surface of the visceral pleura, including fissures, and present a bilateral multifocal distribution. Likewise, there is the presence of a crazy cobblestone pattern, an inverse halo sign, subpleural bands and thickening of the vascular network within the pulmonary anomalies.

### CO-RADS 6

Involves confirmation of the disease by positive RT-PCR [27, 28].

### Differential diagnosis in CT

Within the differential diagnosis, we have viral pneumonia caused by influenza A virus, influenza B virus, respiratory syncytial virus, adenovirus, cytomegalovirus, coronaviruses other than SARS-CoV-2, as well as pneumonia of bacterial etiology [41]. Pneumonia from another viral etiology caused by SARS-CoV-2 is a challenge, since the findings are very similar. However, pneumonias of other viral origin usually present peribronchial or perivascular interstitial inflammation that is directed towards the inner part of the pulmonary interstitium, subpleural and hilar distribution, high attenuation reticular pattern, accentuated fibrous tracts, pulmonary edema, and atelectasis. On the other hand, pneumonia acquired in the bacterial community is characterized by the presence of a segmental or lobar consolidation in the air space, limited by the pleural surface; it may present with ground glass opacities, centrilobular nodules, thickening of the vascular network, and mucus impaction. If you find an immunosuppressed patient [42].

## CT severity index

**Fig. 1:**
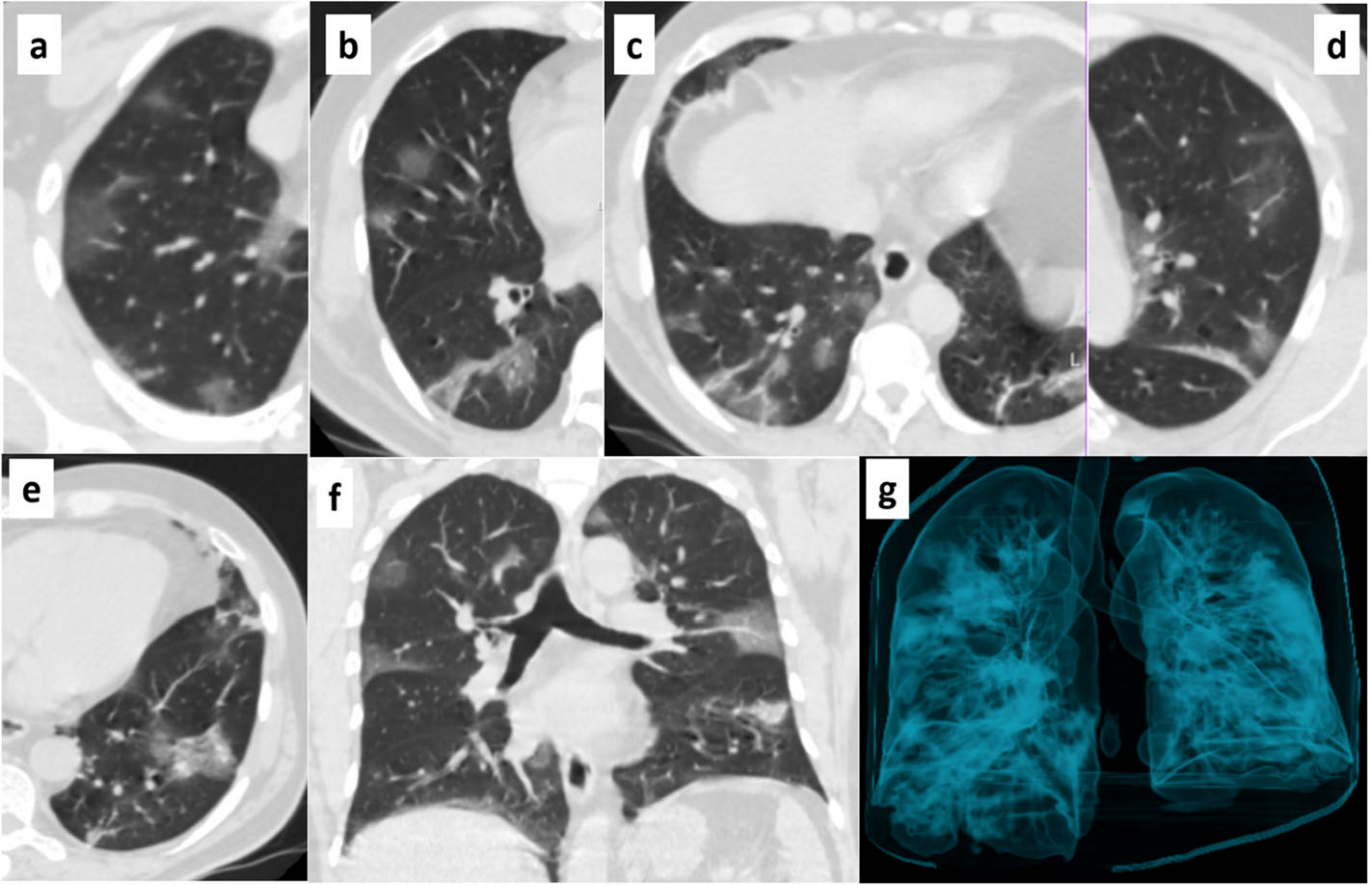
CT severity index of a 51-year-old male patient, progressive stage. **a** Right upper lobe, score 1. **b** Right middle lobe, score 1. **c** Right lower lobe, score 2. **d** Upper left lobe, score 2. **e** Left lower lobe, score 2. **f** Coronal section. **g** Air volumetric reconstruction. The CT severity index is 8

Quantitative evaluation serves to assess the change or progression of pulmonary injury manifested on chest CT. One instrument is the CT severity index. In this, the degree of involvement of each of the five lung lobes is examined and classified into 5 types: no damage (0%), minimal (1–25%), mild (26–50%), moderate (51–75%), or severe (76-100%). No implication corresponds to a lobe score of 0, a minimal implication corresponds to a lobe score of 1, a slight implication is related to a lobe score of 2, a moderate participation concerns a lobe score of 3, and a severe involvement of a lobe score of 4. CT severity index is achieved by adding the results of the 5 lobes (range of possible scores, 0–20) (Fig. 1) [43–45]. Likewise, Li et al. applied the CT severity index in relation to the type of patient COVID-19 in their study: mild, common, and severe-critical. They described that the higher the score, the greater the relationship with the classificatory range of the patients [46]. As days pass from the onset of symptoms, the CT severity index increases, then decreases as improvement is evident [47].

### Artificial intelligence in the covid-19 diagnosis

The use of artificial intelligence algorithms in the radiological field is increasingly common; it is beneficial since it reduces the radiologist’s workload and can reduce the time to diagnose a specific pathology [48]. Its application in the current pandemic is no exception; work is underway on various projects applied to chest CT in patients with COVID-19 [49]. The prototype “CT Pneumonia Analysis2” by the Siemens company stands out.

The CT Pneumonia Analysis2 is an algorithm used for research purposes that identifies and quantifies abnormal tomographic patterns in the lungs automatically on chest CT scans.

This system initially takes noncontrast chest CT to identify and 3D segment the lobes and lungs before documenting abnormalities.

This study produces two combined measures of severity of lung/lobe involvement, quantifying both the extent of COVID-19 abnormalities and the presence of high opacities. The results are used to analyze the severity as well as to document the progress of the disease [50].

### CT changes during the course of disease progression

These manifestations follow a chronological order in the vast majority of cases, with which we can categorize the disease in phases and thus observe the improvement or deterioration of the patient, to solve it [51, 52]. According to Pan et al., we can divide the progression of the disease into 4 radiographic stages, which in turn are related to the patient’s symptoms [7].

#### Early stage (0–4 days)

**Fig. 2:**
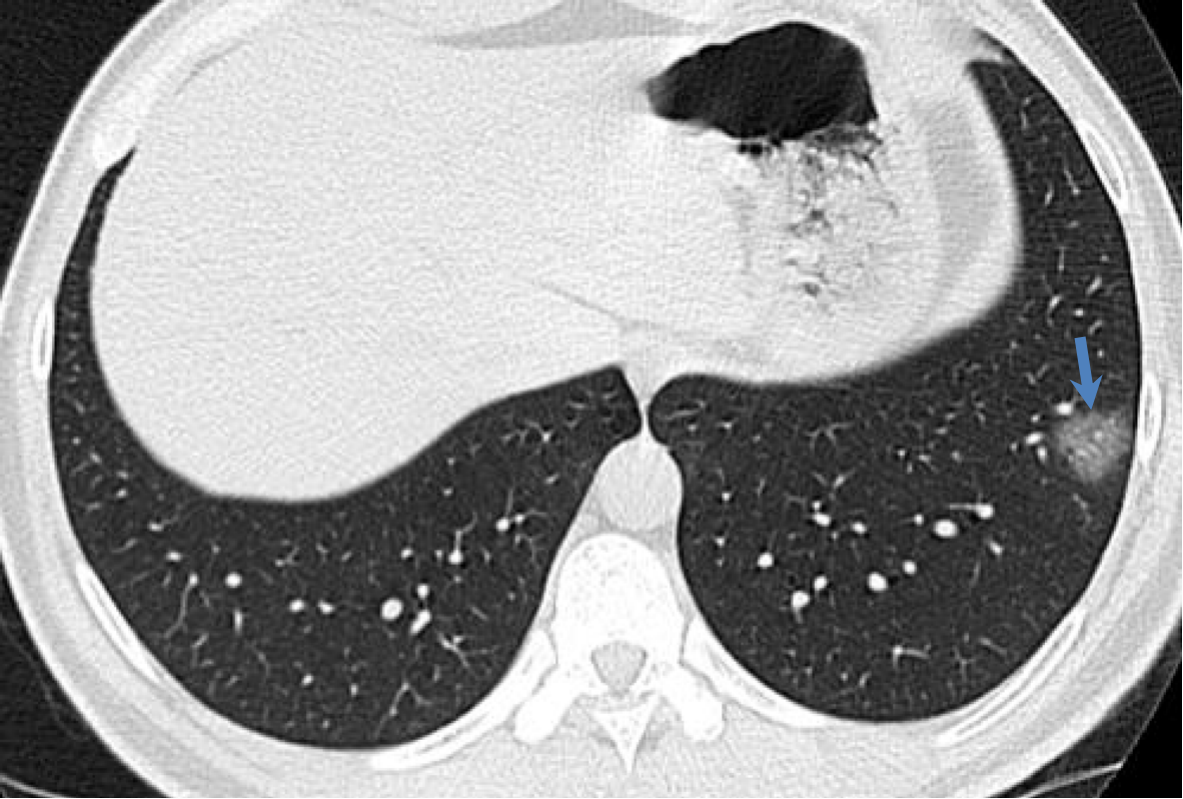
Image of a 36-year-old asymptomatic patient with negative RT-PCR. Pulmonary parenchymal window thin section computed tomography shows ground glass opacity in the left lower lobe of the anterobasal segment of peripheral distribution

It takes place from the onset of symptoms until 4 days later; it is characterized by the presence of an opacity or multiple ground glass opacities distributed subpleurally, generally in the lower lobes, either unilaterally or bilaterally. This is accompanied by vascular accentuation (Fig. 2) [53–55].

#### Progressive stage (5–8 days)

**Fig. 3:**
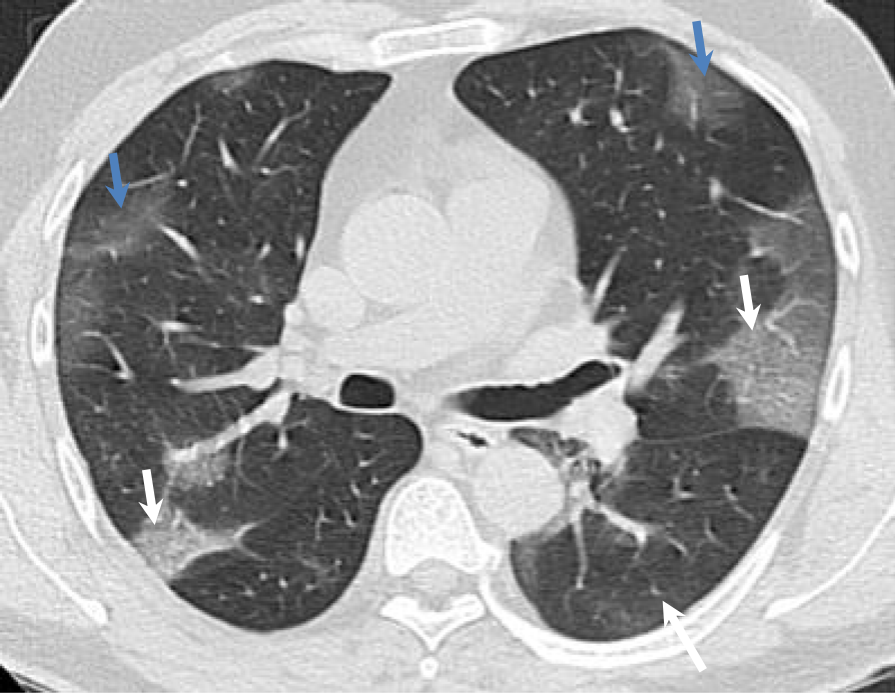
Image of a 49-year-old patient with a history of HIV in treatment with HAART, 6th day of illness with fever and dyspnea. The pulmonary parenchymal window thin section computed tomography shows the presence of ground glass opacities in the bilateral anterosuperior third (blue arrows) and the “crazy stone” pattern of bilateral posterior location (white arrows). There are no consolidations

Within 5–8 days, at this stage, infection progresses rapidly and spreads to multiple lobes, ground glass opacities become diffuse, “crazy cobblestone” pattern appears, and consolidations (Fig. 3) [31].

#### Peak stage (10–13 days)

**Fig. 4:**
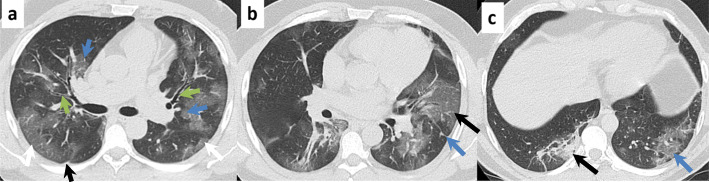
Sequence of images of a 79-year-old patient with fever, dyspnea, and myalgia of 10 days of evolution. The pulmonary parenchymal window thin section computed tomography shows the following: **a** Presence of ground glass opacities in the middle third of both hemithorax, of central distribution (blue arrows) and peripheral distribution (white arrows); parenchymal band declining in the upper third of the left lower lobe (gray arrow), a subpleural band (black arrow) and thickening of the axial peribronchovascular interstitium (green arrows) are added. **b** “Crazy cobblestone” pattern in the lobe of the lingula (black arrow) together with a pleural traction band (blue arrow). **c** Consolidation in the posterior segment of the right lower lobe associated with a parenchymal band in the same segment (black arrow)

It covers from day 9 to day 13; the lung damage slowly reaches its maximum point, the consolidations are fully developed, the air bronchogram sign is observed, a pattern in “crazy paving,” and a greater diffusion of opacities in frosted glass. There are parenchymal bands and subsegmental atelectasis; in some cases, pleural effusion can be witnessed bilaterally (Fig. 4) [38, 56].

#### Absorption stage (≥ 14 days)

**Fig. 5:**
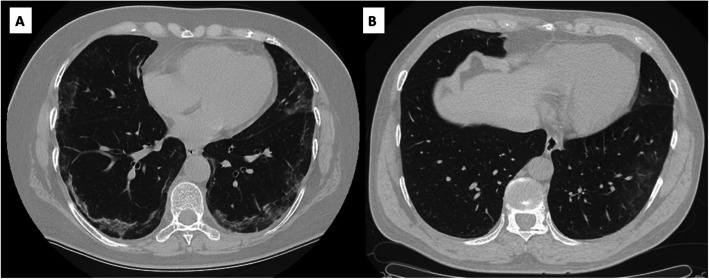
Images of two patients over 50 years old with disease time of 20 days. **a** Parenchymal bands with opacity in ground glass are observed in remission. **b** Observed areas of opacities in ground glass that corresponds to regression areas

It occurs after 14 days of symptom onset and corresponds to disease control. Dissipation of the consolidation is observed; in the same way, it can be observed areas of opacities in ground glass that corresponds to regression areas, and there may also be scarring atelectasis, which indicates fibrosis [57]. As for this phase, the pattern in “crazy paving” is no longer present. In some cases, the course of the disease was short and the transition from the early phase to the absorptive phase was witnessed without intermediaries (Fig. 5) [7, 34, 58, 59].

## Conclusions

In summary, CT is used to monitor the course of the disease since it evaluates the severity of lung involvement. The tomographic characteristic most seen during the stages are bilateral ground glass opacities with a subpleural distribution, which is accompanied by consolidations, a “crazy pavement” pattern, thickening of the vascular network, and air bronchogram, among others. As pneumonia progresses, chest CT provides particular images of COVID-19, with which we can establish, in stages, a sequence of lung injury. The vast majority of patients will show this sequence, especially the first and last stages.

## Data Availability

Being a review article, we do not have data base as the primary studies.

## Abbreviations

CT scan: Computed tomography
RT-PCR: Reverse transcriptase polymerase chain reaction
SARS-CoV-2: Severe acute respiratory syndrome coronavirus type 2
COVID-19: Official name for the disease caused by SARS-CoV-2
